# Ferret brain possesses young interneuron collections equivalent to human postnatal migratory streams

**DOI:** 10.1002/cne.24711

**Published:** 2019-05-15

**Authors:** Justin K. Ellis, Shawn F. Sorrells, Sasha Mikhailova, Manideep Chavali, Sandra Chang, Khalida Sabeur, Patrick Mcquillen, David H. Rowitch

**Affiliations:** ^1^ Eli and Edythe Broad Center of Regeneration Medicine and Stem Cell Research University of California, San Francisco San Francisco California; ^2^ Department of Pediatrics and Neurological Surgery University of California, San Francisco San Francisco California; ^3^ Department of Paediatrics and Wellcome Trust‐MRC Cambridge Stem Cell Institute University of Cambridge Cambridge UK

**Keywords:** cortical interneurons, dorsal migratory stream, doublecortin, ferret, medial migratory stream, posterior migratory stream, postnatal migration, prefrontal cortex, RRID: AB_1640532, RRID: AB839504, RRID:AB_10000320, RRID:AB_10615497, RRID:AB_1079874, RRID:AB_1586992, RRID:AB_2069869, RRID:AB_2194626, RRID:AB_2195374, RRID:AB_2298772, RRID:AB_305869, RRID:AB_477010, RRID:AB_561007, RRID:AB_882455, secretagogin

## Abstract

The human early postnatal brain contains late migratory streams of immature interneurons that are directed to cortex and other focal brain regions. However, such migration is not observed in rodent brain, and whether other small animal models capture this aspect of human brain development is unclear. Here, we investigated whether the gyrencephalic ferret cortex possesses human‐equivalent postnatal streams of doublecortin positive (DCX+) young neurons. We mapped DCX+ cells in the brains of ferrets at P20 (analogous to human term gestation), P40, P65, and P90. In addition to the rostral migratory stream, we identified three populations of young neurons with migratory morphology at P20 oriented toward: (a) prefrontal cortex, (b) dorsal posterior sigmoid gyrus, and (c) occipital lobe. These three neuronal collections were all present at P20 and became extinguished by P90 (equivalent to human postnatal age 2 years). DCX+ cells in such collections all expressed GAD67, identifying them as interneurons, and they variously expressed the subtype markers SP8 and secretagogin (SCGN). SCGN+ interneurons appeared in thick sections to be oriented from white matter toward multiple cortical regions, and persistent SCGN‐expressing cells were observed in cortex. These findings indicate that ferret is a suitable animal model to study the human‐relevant process of late postnatal cortical interneuron integration into multiple regions of cortex.

## Graphical abstract

The human early postnatal brain contains late migratory streams of immature interneurons directed toward prefrontal cortex and other brain regions. We investigated the early postnatal ferret cortex and identified three populations of young neurons with migratory morphology. These three populations expressed GAD67, identifying them as interneurons, and they variously expressed the subtype markers SP8 and secretagogin (SCGN). SCGN+ interneurons appeared to be oriented from white matter toward multiple cortical regions, and persistent SCGN‐expressing cells were observed in cortex. These findings indicate that ferret is a suitable animal model to study the human‐relevant process of late postnatal cortical interneuron integration into multiple regions of cortex.

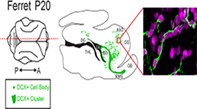

**Abbreviations**
ASG: anterior sigmoid gyrus
BG: basal ganglia
CG: coronal gyrus
DCX: doublecortin
DG: dentate gyrus
DMS: dorsal migratory stream
GAD67: glutamic acid decarboxylase 67
GFAP: glial fibrillary acidic protein
LV: lateral ventricle
MBP: myelin basic protein
MMS: medial migratory stream
OB: olfactory bulb
OCTX: occipital cortex
OG: orbital gyrus
PMS: posterior migratory stream
PSG: posterior sigmoid gyrus
RMS: rostral migratory stream
SCGN: secretagogin
THL: thalamus

## 1 INTRODUCTION

During human brain development, the early postnatal period is characterized by rapid growth and adaptation to the ex utero world. In ill babes, the brain is vulnerable to environmental insults, such as hypoxia‐ischemia and infection, that can result in persistent cognitive deficits and increased risk of psychiatric disease (Cannon, Yolken, Buka, Torrey,, & Collaborative Study Group on the Perinatal Origins of Severe Psychiatric Disorders, 2008; Mittal, Ellman, & Cannon, 2008). Given the importance of this epoch, faithful animal models that mimic key processes of early postnatal human brain development are needed to investigate mechanisms of postnatal cortical circuit formation and the impact of injury.

Recent findings have revealed persistent postnatal streams of late migrating interneurons in human brain up to 12 months of age that are oriented toward specific cortical regions. Humans possess at least three postnatal streams of migrating interneurons; a rostral migratory stream (RMS) that supplies multiple subtypes of interneurons to the olfactory bulbs, which disappears by 12 months postnatally (Sanai et al., 2011), a medial migratory stream (MMS) that branches off of the RMS, putatively supplies calretinin+ interneurons to the ventromedial prefrontal cortex (VMPFC), which disappears by 12 months postnatally (Sanai et al., 2011), and a dorsal migratory “ARC” of cells that is present at the rostral tip of the lateral ventricles, putatively supplies multiple subtypes of interneurons to the anterior cingulate cortex (ACC) and superior frontal gyrus (SFG), and disappears by 5 months postnatally (Paredes et al., 2016).

Although embryonic migration of interneurons to cortex and postnatal migration of interneurons to olfactory bulb (RMA) has been characterized extensively in the mouse, the evidence for postnatal migration of interneurons to cortex is limited. Mice possess a population of postnatal migrating 5HT3a+ cells that exit the RMS up to postnatal Day 10 and terminate in layer six of the orbitofrontal cortex (Inta et al., 2008; Le Magueresse et al., 2011). However, the number of postnatal migrating cells is not as proportionally robust as seen in human and does not reflect human subtype diversity. Furthermore, there is no evidence that mice possess a dorsal migratory stream, seen in human. Finally, the mouse equivalency of human term is considered to be approximately postnatal Day 10, which is when these cells cease migration into the cortex. Therefore, it is unclear if what is observed in mice constitutes true postnatal migration in comparison with human as the phenomenon does not continue past the equivalency of term.

Recent evidence suggests that larger mammalian species, such as rabbits, pigs, sheep, and dolphins, may more closely resemble humans in terms of postnatal migration of interneurons (La Rosa et al., 2018; Luzzati et al., 2003; Morton et al., 2017, but whether this occurs in some small animal species is unclear.). Ferrets have been used as an animal model to study brain development for the past 30 years. Their brain size is significantly increased compared to rodents (Barnette et al., 2009; Raju et al., 2018). Like humans, and unlike rodents, they possess a gyrencephalic cortex (Barnette et al., 2009; Duque & McCormick, 2010; Johnson et al., 2018). Furthermore, they undergo extensive postnatal brain development (McConnell, 1985, 1988; Morton et al., 2017). The ferret also captures other features specific to human brain, such as the presence of an outer sub‐ventricular zone (oSVZ; Gertz, Lui, LaMonica, Wang, & Kriegstein, 2014; Martínez‐Martínez et al., 2016; Reillo & Borrell, 2012). Based on these features, we hypothesized that ferrets would possess postnatal stream of interneurons that were more robust than those seen in rodents.

## 2 MATERIALS AND METHODS

### 2.1 Animals and tissue processing

#### 2.1.1 Animals

Pregnant jills were obtained from Marshall Farms (North Rose, NY) at E26 gestation. All animal experiments were conducted in strict accordance with institutional guidelines and the Guide for the Care and Use of Laboratory Animals, published by the National Institutes of Health. All experiments were approved by the Institutional Animal Care and Use Committee at UCSF.

#### 2.1.2 Tissue processing

Ferret kits were terminally anesthetized using an isoflurane vaporizer and transcardially perfused with 0.1 M phosphate‐buffered saline (PBS) at pH 7.4 followed by 4% paraformaldehyde (PFA) in PBS at pH 7.4. Brains were dissected from the skull and fixed in 4% PFA overnight at 4°C. After fixation, brains were cryoprotected in 30% sucrose in 0.1 M PBS until the brains sunk. For sectioning, brains were frozen to the stage of a sliding microtome (Leica SM2010R). Coronal, sagittal, and horizontal floating sections (50 μm) were cut. Cut sections were stored in 6 well cell culture plates in 0.1 M PBS with azide at 4°C and were used for free floating immunohistochemistry.

### 2.2 Antibody characterization

All relevant antibody information and conditions can be found in Table 1.

The rabbit polyclonal anti‐doublecortin antibody (Cell Signaling Technology, RRID:AB_561007) was characterized by the manufacturer in a Western blot analysis of fetal rat brain tissue lysate, yielding a single protein band at ~45 kDa. We confirmed this in ferret by performing a Western blot analysis of postnatal Day 20 whole brain lysate and observing a similar band. Furthermore, in the current study this antibody specifically stained the cytoplasm of cells with a classic migratory morphology as expected.

The guinea pig polyclonal anti‐doublecortin antibody (Millipore Bioscience Research, RRID:AB_1586992) was characterized by the manufacturer in a Western blot analysis of rat brain tissue lysate, yielding a single protein band at ~45 kDa. We confirmed this in ferret by performing a Western blot analysis of postnatal Day 20 whole brain lysate and observing a similar band. Furthermore, in the current study this antibody specifically stained the cytoplasm of cells with a classic migratory morphology as expected.

The goat polyclonal anti‐GAD67 antibody (Abcam, RRID: AB_1640532) was characterized by the manufacturer in Western blot analyses of both mouse and human brain lysate, yielding a single protein band of 70 kDa. We confirmed this in ferret by performing a Western blot analysis of postnatal Day 20 whole brain lysate and observing a similar band.

The mouse monoclonal anti‐GFAP antibody (Sigma Aldrich, RRID:AB_477010) was characterized by the manufacturer by performing a Western blot analysis of whole extract of rat B35 cells, yielding a band of ~50 kDa. We confirmed this in ferret by performing a Western blot analysis of postnatal Day 20 whole brain lysate and observing a similar band.

The mouse monoclonal anti‐Olig2 antibody (gift from Chuck Stiles lab) was previously characterized by Lu et al., Neuron, 2000 and identifies oligodendrocytes. We further characterized this antibody in ferret by performing a Western blot analysis of postnatal Day 20 whole brain lysate and observing a band at 57 kDa, as expected.

The goat polyclonal anti‐Sox10 antibody (Santa Cruz Biotechnology, RRID:AB_2195374) was characterized by the manufacturer by performing a Western blot analysis on both nontransfected and mouse and human recombinant transfected 293T whole cell lysates, yielding a single protein band at 55 kDa. We characterized this antibody in ferret by performing a Western blot analysis of postnatal Day 20 whole brain lysate and observing a similar band.

The rabbit polyclonal anti‐Iba1 antibody (Wako Chemicals, RRID: AB_839504) was characterized by the manufacturer by performing a Western blot on isolated rat microglia, yielding an appropriate band at 17 kDa.

The goat polyclonal anti‐Sp8 antibody (Santa Cruz Biotechnology, RRID:AB_2194626) was characterized by Radonjic et al., Front Neuroanat, 2014 who performed a Western blot on human fetal cerebral cortex, yielding a band at ~50 kDa. We characterized this antibody by performing a Western blot on postnatal Day 20 ferret whole brain lysate and observing a similar band.

The rabbit polyclonal anti‐Secretagogin antibody (Sigma Aldrich, RRID:AB_1079874) was characterized by Sanaganarapu et al, PLOS ONE, 2016 by performing a Western blot on BRIN‐BD11 insulinoma cell lysates, yielding a single protein band of ~32 kDa. We characterized this antibody by performing a Western blot analysis on postnatal Day 20 ferret whole brain lysate and observing a similar band.

The mouse monoclonal anti‐calretinin antibody (Swant, RRID:AB_10000320) was characterized by the manufacturer by performing immunohistochemical stains on wild type and calretinin knock out mice.

The rabbit monoclonal anti‐Cleaved Caspase 3 antibody (Cell Signaling, RRID:AB_2069869) was characterized by the manufacturer by performing Western blot analyses on control and cytochrome c‐treated HeLa, NIH/3T3, and C6 cells and observing two protein bands at 17 and 19 kDa.

The rat monoclonal anti‐myelin basic protein antibody (Abcam, RRID:AB_305869) was characterized by the manufacturer by performing a Western blot analysis on mouse brain lysate, yielding two isoforms at 19 and 26 kDa.

The mouse monoclonal NeuN antibody (Millipore, RRID:AB_2298772) was characterized by the manufacturer by performing Western blot analyses on control brain tissue and observing 2–3 bands in the 46–48 kDa range.

The mouse monoclonal SATB2 antibody (Abcam, RRID:AB_882455) was characterized by the manufacturer by performing Western blot analyses on control HT1080 and NIH/3T3 cells and observing one protein band at 85 kDa.

The chicken polyclonal TBR1 antibody (Millipore, RRID:AB_10615497) was characterized by the manufacturer by performing Western blot analyses on human fetal lysate and observing one protein band at 68 kDa.

### 2.3 Immunohistochemistry, confocal and epifluorescent microscopy, and image processing

#### 2.3.1 Staining

All doublecortin, GAD67, SATB2, and TBR1 immunohistochemistry was performed using tyramide signal amplification. In short, free‐floating sections (50 μm) were first mounted on glass slides and allowed to dry for 1 hr. Slides were then rinsed in PBS, and antigen retrieval was performed at 95°C in 0.01 M Na Citrate buffer, pH = 6. Durations of antigen retrieval can be found in Table 1. Following antigen retrieval, slides were washed with PBS‐T (0.2% TX100 in PBS) for 15 min, incubated with 0.02 N HCl in PBS for 30 min at room temperature (RT), and then blocked with TNB solution (0.1 M Tris–HCl, pH 7.5, 0.15 M NaCl, 0.5% blocking reagent from PerkinElmer) for 1 hr at RT. Slides were incubated in primary antibodies overnight at 4°C (see Table 1). All antibodies were diluted in TNB solution. The following day, slides were rinsed in PBS‐T for 1 hr at RT and then incubated with biotinylated secondary antibodies (1:500; Jackson Immunoresearch Laboratories), as well as any Alexa secondaries being used (1:750), for 1 hr at room temperature. Sections were then incubated for 30 min in streptavidin‐horseradish peroxidase that was diluted (1:200) with TNB. Finally, sections were incubated in tyramide‐conjugated fluoroscein for 5 min at 1:100. Slides were then covered with glass cover slips using DAPI Fluoromount‐G (Thermo Fisher Scientific).

**Table 1:** Description of antibodies used in this study

Antibody	Immunogen	Source, Catalog#, RRID, species/clonality	Concentration, antigen retrieval
Doublecortin (DCX)*	Synthetic peptide corresponding to human doublecortin	Cell Signaling Technology, Cat# 4604, RRID:AB_561007, rabbit polyclonal	1:500, AR 5 min
Doublecortin (DCX)*	Synthetic peptide (YLPLSLDDSDSLGDSM) at the carboxy terminus of mouse and rat doublecortin	Millipore Bioscience Research, Cat# AB2253, RRID:AB_1586992, guinea pig polyclonal	1:500, AR 5 min
Glutamic acid decarboxylase 67 (GAD67)*	Synthetic peptide (C‐PDSPQRREKLHK) corresponding to internal sequence amino acids 526‐537 of Human GAD67 (NP_000808.2)	Abcam, Cat# ab80589, RRID: AB_1640532, goat polyclonal	1:500, AR 30 min
Glial Fibrillary acidic protein (GFAP)	Purified from pig spinal cord	Sigma Aldrich, Cat# G3893, RRID:AB_477010, mouse monoclonal IgG1 (Clone G‐A‐5)	1:400
Olig2		gift from Chuck Stiles Lab, mouse monoclonal	1:200
Sox10	Epitope mapping at the N terminus of human Sox10	Santa Cruz Biotechnology, Cat# sc‐17342, RRID:AB_2195374, goat polyclonal IgG (Clone N‐20)	1:100, AR 5 min
Iba1	Synthetic peptide corresponding to C terminus of Iba1	WAKO, Cat# 019‐19741, RRID: AB_839504, rabbit polyclonal	1:500
Sp8	Synthetic peptide of the carboxy terminus of human Sp8	Santa Cruz Biotechnology, Cat# sc‐104661, RRID:AB_2194626, goat polyclonal	1:1000, AR 5 min
Secretagogin (SCGN)	Secretogoggin recombinant protein epitope signature tag	Sigma Aldrich, Cat# HPA006641, RRID:AB_1079874, rabbit polyclonal	1:1000
Calretinin	Produced in mice by immunization with recombinant human calretinin‐22k	Swant, Cat# 6B3, RRID:AB_10000320, mouse monoclonal	1:500
Cleaved Caspase3	Synthetic peptide corresponding to amino‐terminal residues adjacent to Asp175 of human caspase‐3	Cell Signaling, Cat# 9669, RRID:AB_2069869, rabbit monoclonal IgG (Clone D175)	1:200
Myelin basic protein (MBP)	Synthetic peptide (DENPVV) corresponding to amino acids 82–87 of the cow MBP	Abcam, Cat# AB7349, RRID:AB_305869, rat monoclonal	1:500
NeuN	Purified cell nuclei from mouse brain	Millipore, Cat# MAB377, RRID:AB_2298772, mouse monoclonal IgG1 (Clone A60)	1:200
SATB2*	Recombinant fragment corresponding to human SATB2 C‐terminal	Abcam, Cat#51502, RRID:AB_882455, mouse monoclonal	1:500
TBR1*	KLH‐conjugated linear peptide corresponding to mouse TBR1	Millipore, Cat#AB2261, RRID:AB_10615497, chicken polyclonal	1:100

* Tyramide signal amplification (TSA) was required.

Primary antibodies that did not require tyramide signal amplification were stained in the following manner. Free floating sections (50 μm) were first mounted on glass slides and allowed to dry for 1 hr. Slides were then rinsed in PBS, and antigen retrieval was performed at 95°C in 0.01 M Na Citrate buffer, pH = 6. Durations of antigen retrieval can be found in Table 1. Following antigen retrieval, slides were washed with PBS‐T (0.2% TX100 in PBS) for 15 min, blocked for 1 hr in blocking solution (10% goat or donkey serum in 0.1 M PBS w/ 0.2% Triton‐X) and incubated with primary antibody in blocking solution overnight at 4°C. Sections were washed six times for 10 min each in PBS‐T on a rocker at room temperature. Sections were then incubated for 2 hr with Alexa secondary antibody in blocking solution (1:750). Sections were then washed six times for 10 min each in washing solution. Sections were covered with cover slips using DAPI Fluoromount‐G.

#### 2.3.2 Confocal and epifluorescent microscopy

All confocal images were taken using a Leica Sp8 confocal microscope (Leica Microsystems, Wetzlar, Germany). Images were acquired as 2um thick optical sections.

Tile scans used for mapping doublecortin signal were taken using a Zeiss Axioimager (Carl Zeiss, Oberkochen, Germany).

#### 2.3.3 Image processing

Lif files were covered to tif files using ImageJ. All image files were imported into Photoshop CS6 and adjusted for brightness and contrast only.

Tile scans of DCX stained P20 ferret brain sections in the sagittal, coronal, and horizontal planes were uploaded into Adobe Illustrator CS6. The outline of each section was traced using the pen tool. DCX+ cell bodies exhibiting an elongated morphology characteristic of a migrating cell were identified and marked with an individual dot.

### 2.4 Cell counting and quantification

#### 2.4.1 DCX+ cluster quantifications

DCX+ clusters between the ventricle and each of the three streams were compared at P20, P40, P65, and P90. Confocal images of each cluster were uploaded into ImageJ. The anterior and posterior borders of each cluster were visually determined, and a line was drawn representing the maximum possible length of each cluster. Three evenly spaced sections from each of three animals were included for each stream and each time point. Distances in micrometers were recorded, and one way ANOVAs were performed on the average distance taken for each animal using Prism version 6, Graphpad. Significance was set at *p* = .05.

#### 2.4.2 DCX+ cell densities

Confocal images were obtained for each stream proximal to the DCX+ cluster. Images were taken at P20, P40, P65, and P90. Three sections from each of three animals were included for each stream and each time point. Images were loaded into ImageJ, and DCX+ cell bodies were counted. The DCX+ cell density was calculated by dividing the number of cells per section by the area of the section multiplied by the tissue thickness (50 μm). Student's *t*‐tests were performed on the average number of cells per animal using Prism version 6, Graphpad. Significance was set at *p* = .05.

#### 2.4.3 DCX co‐localizations

Confocal images were taken of the MMS in the sagittal plane at the indicated ages and were loaded into ImageJ. Percent co‐localizations were calculated by counting the number of DCX+ cell bodies per image and dividing by the number of co‐localized cells. Three sections from each of three animals were counted.

#### 2.4.4 White matter cells and caspase+ cells

Sagittal sections of the MMS were stained with either secretagogin or cleaved caspase 3 at the indicated ages and were visually inspected using a confocal microscope. SCGN+ cells in the white matter with a mature, differentiated morphology were counted manually due to low density. All SCGN+ cells in the MMS were included in each count. Cleaved caspase 3+ cells in the white matter were similarly counted manually. All positive cells in the MMS were included in each count. Three sections from each of three animals were counted. Student's *t*‐tests were performed to determine significance using Prism version 6, Graphpad.

### 2.5 Tissue clearing and staining with iDISCO+

The iDISCO+ protocol for clearing thick tissue sections was performed as described (Renier et al., 2016). In brief, ferrets were transcardially perfused at P20 and postfixed O/N. Brains were extracted and cut in half. Individual hemispheres were stored in PBS azide until ready to be utilized. Fixed samples were washed in PBS for 1 hr twice, then in 20% methanol (in ddH_2_O) for 1 hr, 40% methanol for 1 hr, 60% methanol for 1 hr, 80% methanol for 1 hr, and 100% methanol for 1 hr twice. Samples were then bleached with 5% H_2_O_2_ (1 volume of 30% H_2_O_2_ for five volumes of methanol, ice cold) at 4°C overnight. After bleaching, samples were re‐equilibrated at room temperature slowly and re‐hydrated in 80% methanol in H_2_O for 1 hr, 60% methanol/H_2_O for 1 hr, 40% methanol/H_2_O for 1 hr, 20% methanol/H_2_O for 1 hr, and finally in PBS/0.2% TritonX‐100 for 1 h twice. Pretreated samples were then incubated in PBS/0.2% TritonX‐100/20% DMSO/0.3 M glycine at 37°C for 36 hr, then blocked in PBS/0.2% TritonX‐100/10% DMSO/6% Donkey Serum at 37°C for 2 days. Hemispheres were then incubated in primary antibody dilutions of 1:100 in PBS‐Tween 0.2% with Heparin 10 μg/mL (PTwH)/5% DMSO/3% Donkey Serum at 37°C for 7 days. Primary antibody solutions were changed every other day. Samples were then washed in PTwH for 24 hr (five changes of the PTwH solution over that time), then incubated in secondary antibody dilutions (e.g., donkey anti‐rabbit‐Alexa647 at 1:500 in PTwH/3% Donkey Serum) at 37°C for 4 days. Samples were finally washed in PTwH for 1d before clearing and imaging.

Immunolabeled brains were cleared with the following method. Samples were dehydrated in 20% methanol (in ddH_2_O) for 1 hr, 40% methanol/H_2_O for 1 hr, 60% methanol/H_2_O for 1 hr, 80% methanol/H_2_O for 1 hr, and 100% methanol for 1 hr twice. Samples were incubated overnight in 1 volume of methanol/2 volumes of dichloromethane (DCM, Sigma 270997‐12X100ML) until they sank to the bottom of the vial (50 mL falcon tubes were used throughout the process). The sample was then washed for 20 min twice in 100% DCM. Finally, samples were incubated (without shaking) in DiBenzyl Ether (DBE, Sigma 108014‐1KG) until clear (about 30 min) and stored in DBE at room temperature.

#### 2.5.1 Light sheet and spinning disk microscopy

Cleared samples were imaged on a light‐sheet microscope (Custom built AZ‐100 by Nikon Imaging Center, UCSF) equipped with a sCMOS camera (Andor Neo) and a ×2 (WD = 45 mm, N.A. = 0.2) or ×5 (WD = 15 mm, N.A. = 0.5) objective lens. Scans were made at either a ×1 or ×2 zoom magnification. The samples were scanned with a step‐size of 16 μm (×2 Obj.) or 2.3 μm (×5 obj.).

Higher magnification images were taken of each sample using the ×10 objective on a spinning disk microscope (Yokagawa CSU22).

#### 2.5.2 Image processing

All image and movie files were processed and snapshots taken using Imaris v.7.22. Brightness and contrast were the only attributes adjusted.

## 3 RESULTS

### 3.1 Postnatal DCX+ cell collections in the ferret brain diminish with increasing postnatal age

**Figure 1:**
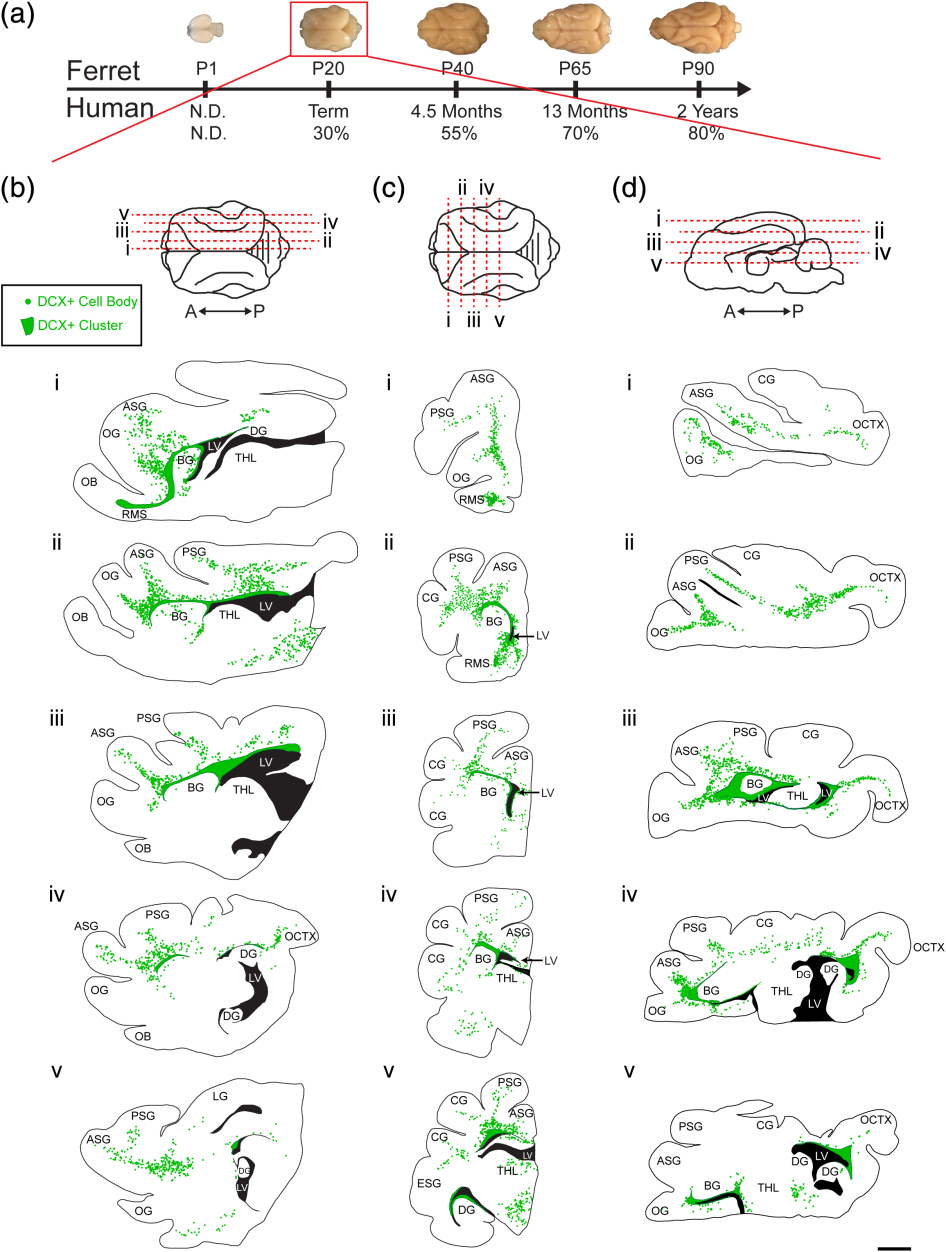
Ferrets at postnatal Day 20 (P20) have a widespread population of doublecortin (DCX+) positive cells. (a) Comparison of ferret and human developmental timelines. Using percent maximum brain weight, ferret brain development was aligned with human to determine comparative ages. At P20, ferrets are 30% of their maximum brain weight. (b–d) Maps of DCX+ cells with migratory morphology in the P20 ferret. Sagittal (b), coronal (c), and horizontal orientations (d). Scale bar = 1 mm [Color figure can be viewed at http://wileyonlinelibrary.com]

**Figure 2:**
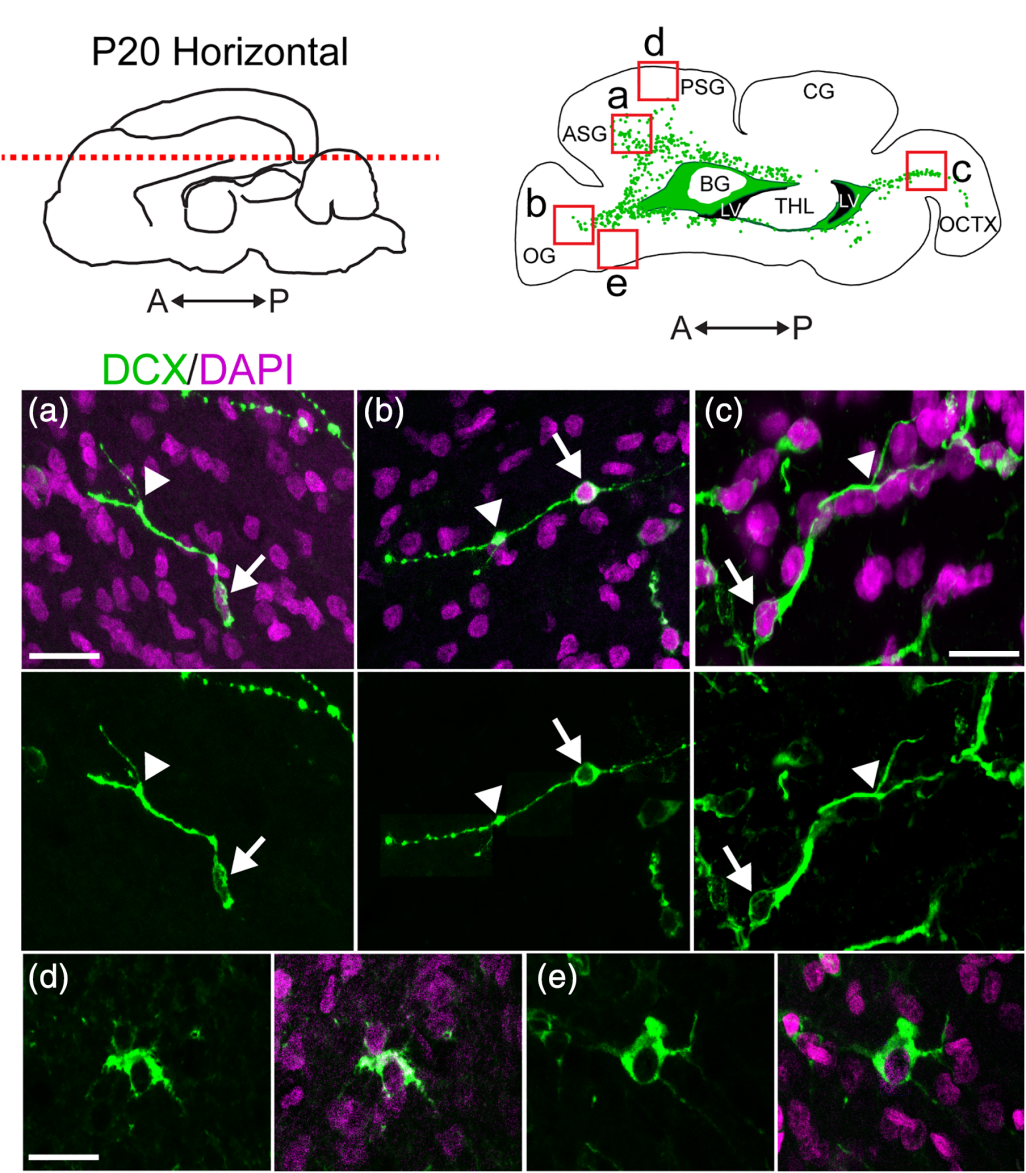
DCX+ cells in the P20 ferret cortex and white matter exhibit different morphologies. (a–c) DCX+ cells in the white matter exhibit a migratory morphology including an elongated cell body (arrow) and a single, sometimes forked, leading process (arrowhead). (d,e) DCX+ cells in the cortex have large, rounded cell bodies, large nuclei, and multiple extended processes. Scale bar a, b, d, e = 10 μm. Scale bar c = 7.5 μm [Color figure can be viewed at http://wileyonlinelibrary.com]

To study the distribution of migratory neurons in the ferret brain, we began by staining for the immature neuronal marker doublecortin (DCX) at postnatal Day 20 (P20). At this age, the ferret brain is equivalent to the term human brain in its percentage of maximum brain volume (Figure 1a; Workman, Charvet, Clancy, Darlington, & Finlay, 2013). At P20, we found many individual DCX+ cells distributed throughout the white matter and cortex. We also observed dense clusters of DCX+ cells surrounding the lateral ventricles. To visualize the location and orientation of these DCX+ cells, we created maps of the individual and clustered DCX+ cells in the P20 ferret brain in the coronal, sagittal, and horizontal planes (Figure 1b–d). Individual DCX+ cells were relatively abundant in dorsal, compared to ventral, brain regions and in several white matter tracts. White matter tracts with abundant DCX+ cells were associated with sections of DCX+ clusters containing protrusions of cells into the white matter. At P20, DCX+ cells in the white matter in anterior, dorsal, and posterior gyri all had features of migratory neurons. These individual cells had an elongated cell body and a forked leading process (Figure 2a,b,c). In contrast, DCX+ cells in the cortex had large round cell bodies and multiple processes (Figure 2d,e). Cortical DCX+ cells were excluded from the maps as they did not have characteristics of migratory cells. These findings indicate that postnatal ferret brain possesses a widespread distribution of DCX+ cells with a migratory morphology oriented toward specific cortical regions.

### 3.2 A young neuron collection—equivalent to human MMS—is present in the postnatal ferret frontal cortex

**Figure 3:**
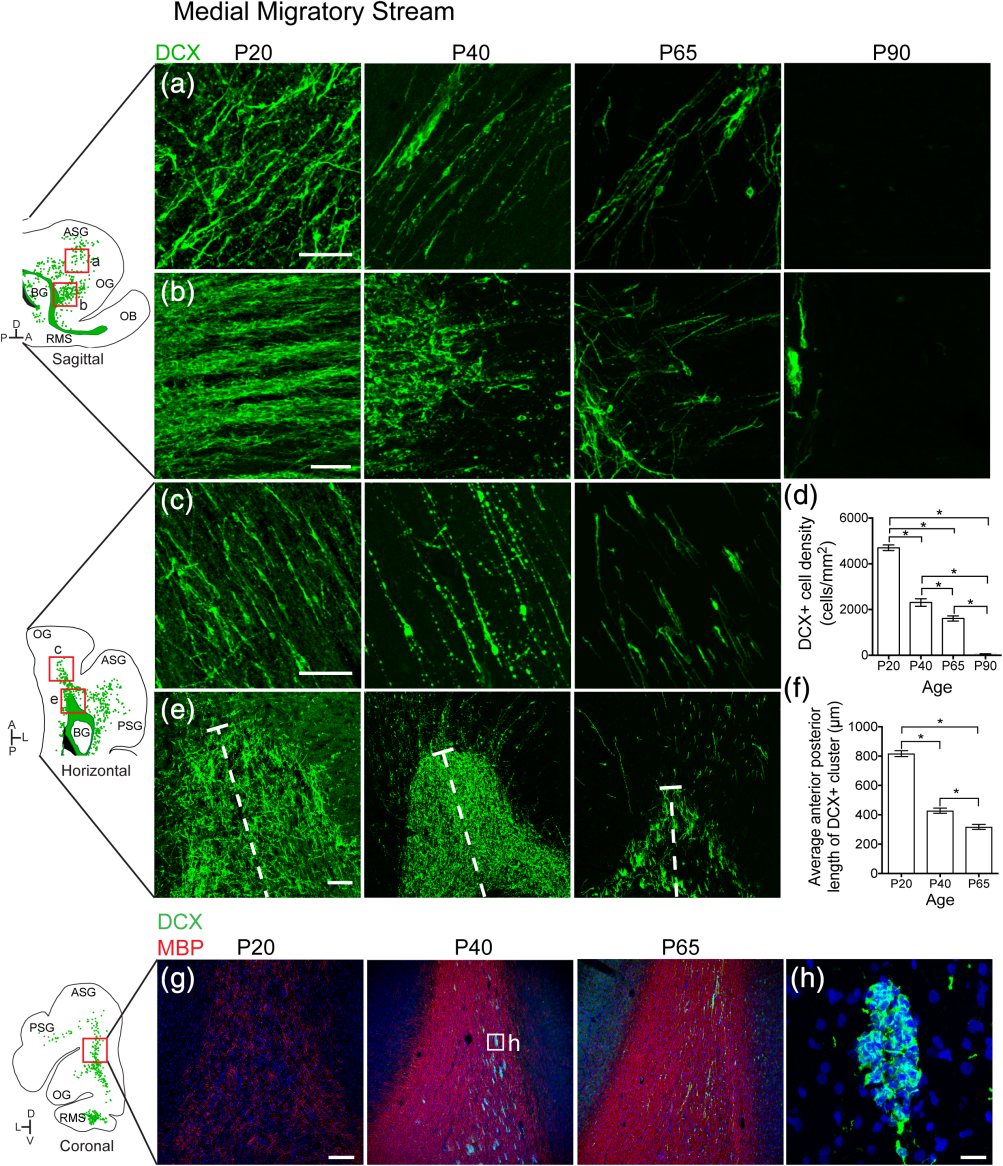
The ferret medial migratory stream condenses into clusters and disappears between P65 and P90. (a,b) P20 sagittal section highlighting the MMS. (a) Individual DCX+ cells with migratory morphology oriented away from the RMS and toward the PFC. (b) DCX+ chains protruding from the RMS. (c,e) P20 horizontal section. (c) Individual DCX+ cells. (d) Quantification of the decrease in DCX+ cell density over time. (e) DCX+ cluster anterior to the lateral ventricle. (f)Quantification of the decrease in DCX+ cluster size over time. (g) Sections at P20, P40, and P65 showing increased density of myelin basic protein (MBP) and emergence of DCX+ clusters. (h) High resolution image of DCX+ cluster. Scale Bar a,c = 50 μm. Scale bar b = 40 μm. Scale bar e = 25 μm. Scale bar g = 200 μm. Scale bar h = 20 μm [Color figure can be viewed at http://wileyonlinelibrary.com]

To determine whether ferret possesses medial, rostral, and dorsal migratory streams, like in human, we next investigated the organization and possible contribution of white matter DCX+ cells to specific cortical regions in anterior, dorsal and posterior gyri. The most prominent group of DCX+ cells was present on the anterior side of the RMS‐like structure (Figure 3). At P20, many individual DCX+ cells (Figure 3a,c) were oriented parallel to one another in the white matter tracts of the orbital gyrus (OG) and anterior sigmoid gyrus (ASG), which together make up the ferret prefrontal cortex. In conjunction with the individual cells, chains of tightly‐associated DCX+ cells (Figure 3b) were observed extending anteriorly out of the RMS toward the PFC. To investigate changes in these populations with age, we next looked at animals aged P40–P90. The DCX+ cluster from which the chains protruded was best visualized in the horizontal plane **(**Figure 3e). At older ages, the DCX+ cells in this region decreased significantly in number (Figure 3d). Individual cells in the white matter tract of the PFC were still present at P65, but were absent by P90. Correlating with this observation, the chains of DCX+ cells were more fragmented at P40, and were not observed after P65. The DCX+ cluster between the ventricle and migratory cells also decreased significantly in size between P20 and P65 (Figure 3f). Interestingly, the timing of the decrease of DCX+ chains protruding from the RMS coincided with an increase in MBP in the surrounding white matter **(**Figure 3g,h). We also observed that as MBP staining increased from P20 to P65, individual DCX+ cells in the stream condensed into clusters in the white matter. These findings indicate that the ferret possesses postnatal young neuron collections similar to human RMS and MMS.

### 3.3 A young neuron collection oriented toward postnatal ferret dorsomedial cortex

**Figure 4:**
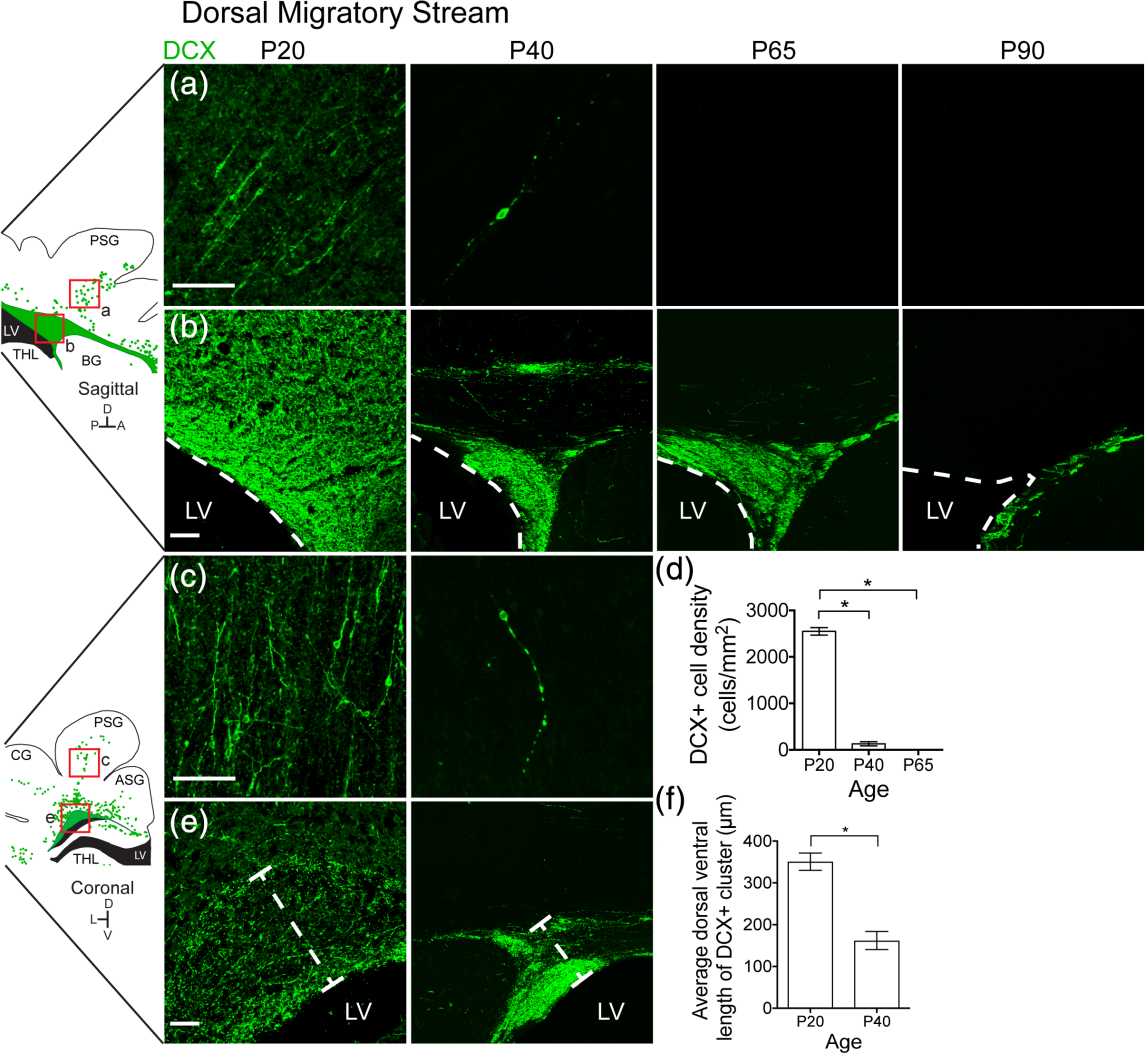
The ferret dorsal migratory stream mostly disappears by P40. (a,b) P20 sagittal section highlighting the DMS. (a) Individual DCX+ cells with migratory morphology oriented away from the dorsal lateral ventricle and toward the PSG. (b) DCX+ cluster dorsal to the lateral ventricle. (c,d) P20 coronal section highlighting the DMS. (c) Individual DCX+ cells with migratory morphology oriented dorsally toward the PSG. (d) Quantification of the decrease in DCX+ cell density over time. (e) DCX+ cluster dorsal to the lateral ventricle. (f) Quantification of the decrease in DCX+ cluster size over time. Scale bar a–c,e = 100 μm [Color figure can be viewed at http://wileyonlinelibrary.com]

We next studied other gyri containing DCX+ migratory neurons at P20 to see if these populations disappeared at a uniform rate. At P20 there is a prominent dorsal population of DCX+ cells in the white matter of the posterior sigmoid gyrus (PSG; Figure 4 ). Individual cells with migratory morphology were observed in the white matter of the PSG oriented away from the lateral ventricle and toward the cortex (Figure 4a). A large DCX+ cluster was also observed adjacent to the dorsolateral ventricle (Figure 4b). In contrast to the MMS, we did not detect discrete chains of DCX+ cells exiting the dense DCX+ cluster at the dorsolateral ventricles. In the coronal plane, the same stream was found to occupy the white matter tracts of both the PSG and the ASG. (Figure 4c). By P40, the individual cells in this region had already become rare. A few scattered DCX+ cells were observed, but by P65 they had disappeared completely (Figure 4d). By P40, the large population of DCX+ cells adjacent to the ventricle had decreased significantly in size, correlating in timing with the decrease in individual DCX+ cells (Figure 4e,f). Compared to the frontal lobe stream, the dorsal population of DCX+ cells diminished more quickly during early postnatal life. Individual DCX+ cells also did not coalesce into clusters with increasing age and MBP condensation, like those seen in the MMS (data not shown). These findings indicate that the ferret possesses a postnatal stream similar to the human DMS “ARC.”

### 3.4 Evidence that a posterior interneuron collection oriented toward the postnatal ferret occipital cortex comprises a novel migratory stream

**Figure 5:**
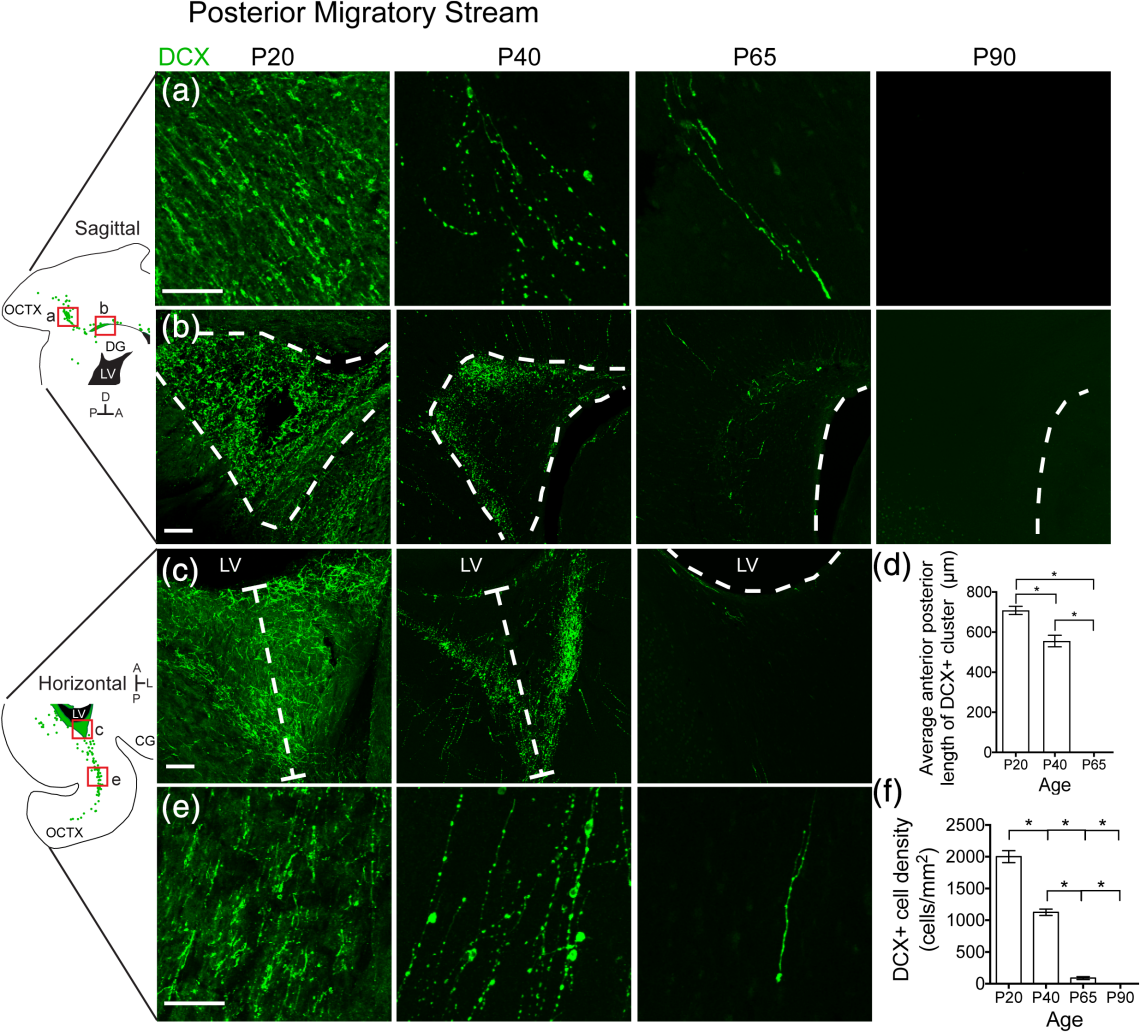
The ferret posterior migratory stream disappears between P40 and P65. (a,b) P20 sagittal section highlighting the PMS. (a) Individual DCX+ cells with migratory morphology oriented away from the lateral ventricle and toward the occipital cortex. (b) DCX+ cluster posterior to the lateral ventricle. (c,e) P20 horizontal section highlighting the PMS. (c) DCX+ cluster posterior to the lateral ventricle. (d) Quantification of the decrease in DCX+ cluster size over time. (e) Individual DCX+ cells with migratory morphology oriented away from the lateral ventricle and toward the occipital cortex. (f) Quantification of the decrease in DCX+ cell density over time. Scale bar a–c,e = 100 μm [Color figure can be viewed at http://wileyonlinelibrary.com]

Having confirmed young neuron collections that mimic three streams found in the human postnatal brain, we used ferret as a means to discover putative streams in other brain regions. In addition to the anterior (RMS, MMS) and dorsal (DMS) streams, we observed a novel population of DCX+ cells in the posterior white matter of the P20 ferret oriented toward the occipital cortex (Figure 5a,e). Individual cells appeared to be oriented away from a dense cluster of DCX+ cells next to the posterior lateral ventricle (Figure 5b,c). Like the DMS, but unlike the MMS, no chains of DCX+ cells were observed exiting this cluster, nor did cells coalesce into clusters in the white matter with progressing age (data not shown). Individual cells in this region persisted slightly later into postnatal life than in the dorsal stream and were observed until P65 (Figure 5f). By P90 we no longer detected them in the posterior cortex. The DCX+ cluster began to decrease in size after P20 and had disappeared completely by P65 (Figure 5d). We next investigated the types of cells that comprise these late postnatal populations in the ferret.

### 3.5 Postnatal DCX+ cells with migratory morphology are young interneurons

**Figure 6:**
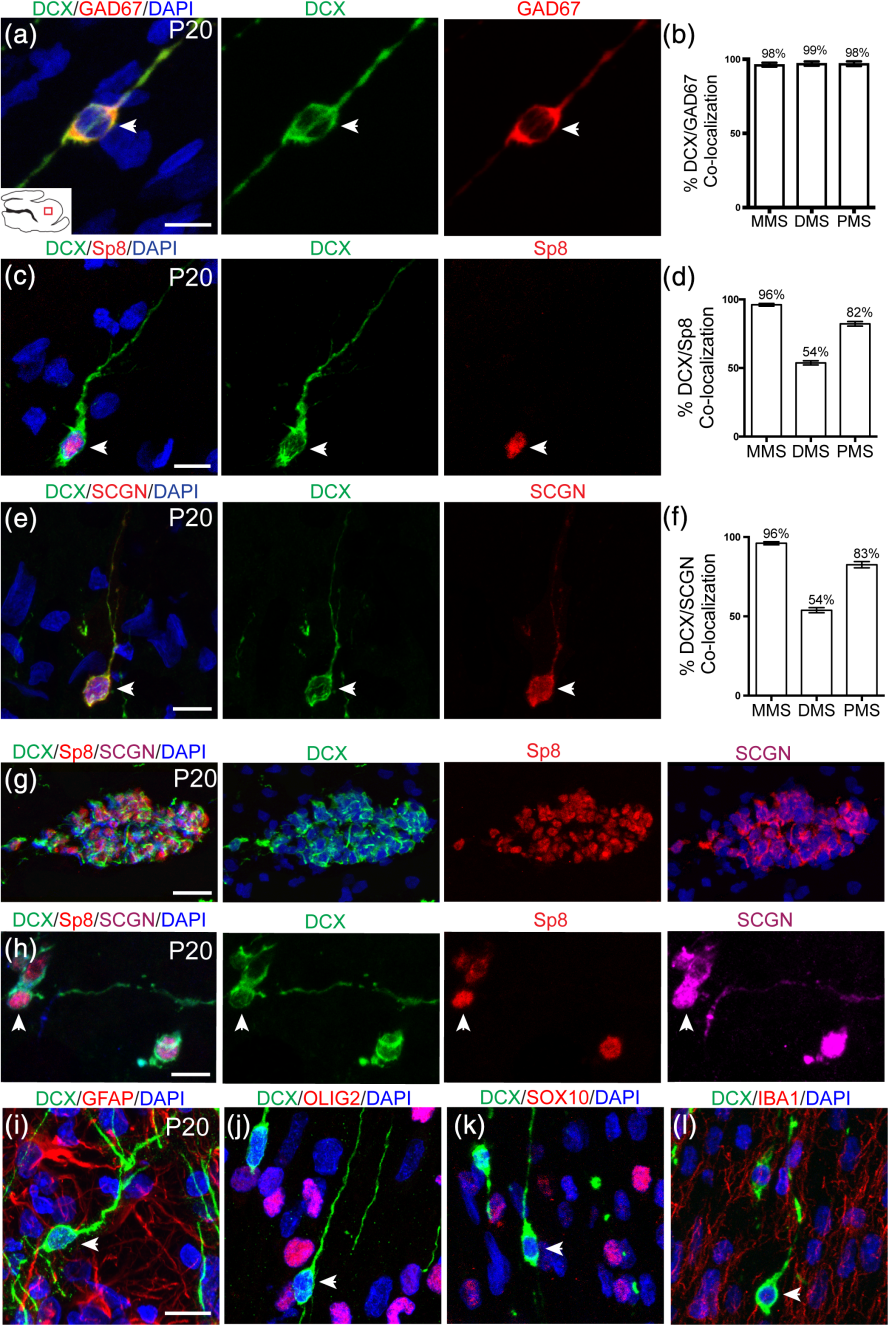
DCX+ cells in postnatal streams are interneurons. (a,c,e) P20 DCX+ cells in each stream express GAD67, Sp8, and SCGN, respectively. (b) Quantification of DCX/GAD67 co‐localization in each stream. (d) Quantification of DCX/Sp8 co‐localization in each stream. (f) Quantification of DCX/SCGN co‐localization in each stream. (g) Individual clusters express DCX, Sp8, and SCGN. (h) Individual DCX+ cells express both Sp8 and SCGN. (i–l) DCX+ cells do not co‐localize with glial markers GFAP, OLIG2, SOX10, or IBA1. Scale bar (a–l) = 10 μm. Scale bar g = 50 μm [Color figure can be viewed at http://wileyonlinelibrary.com]

To further characterize the DCX+ cells, we stained them for markers of neurons and glial cells (Figure 6). At P20, cells in each stream co‐expressed GAD67 and DCX to a very high degree, suggesting that they are likely young inhibitory interneurons (Figure 6a,b). DCX+ cells in each stream also expressed Sp8, further suggesting they are interneurons (Figure 6c). Each stream did not co‐express DCX and Sp8 to the same degree, with the MMS expressing the most and the DMS expressing the least (Figure 6d). Interestingly, DCX+ cells were also found to express SCGN, a calcium binding protein that is expressed in human cortical interneurons but not rodent cortical interneurons (Raju et al., 2018; Figure 6e). The degree of co‐localization between DCX and SCGN was almost identical to that of DCX and Sp8 (Figure 6f). This led us to triple stain both individual cells and clusters for further verification of their identity (Figure 6g,h). Clusters observed in P20 sagittal sections of the MMS were positive for DCX, SCGN, and Sp8 to some degree. Finally, we wanted assess whether any DCX+ cells expressed glial markers, as has been shown (Boulanger & Messier, 2017; Liu et al., 2018). DCX+ cells did not express markers of glial cells, such as GFAP (Figure 6i), a marker of astrocytes, OLIG2 (Figure 6j), and SOX10 (Figure 6k), markers of oligodendrocytes, or Iba1 (Figure 6l) a marker of microglial cells. From these data, we concluded that DCX+ cells with a migratory morphology were young interneurons. The observation that SCGN is expressed in these cells led us to question whether or not they could be integrated into cortical circuitry.

### 3.6 SCGN+ cells appear to transition from the white matter to the cortex

**Figure 7:**
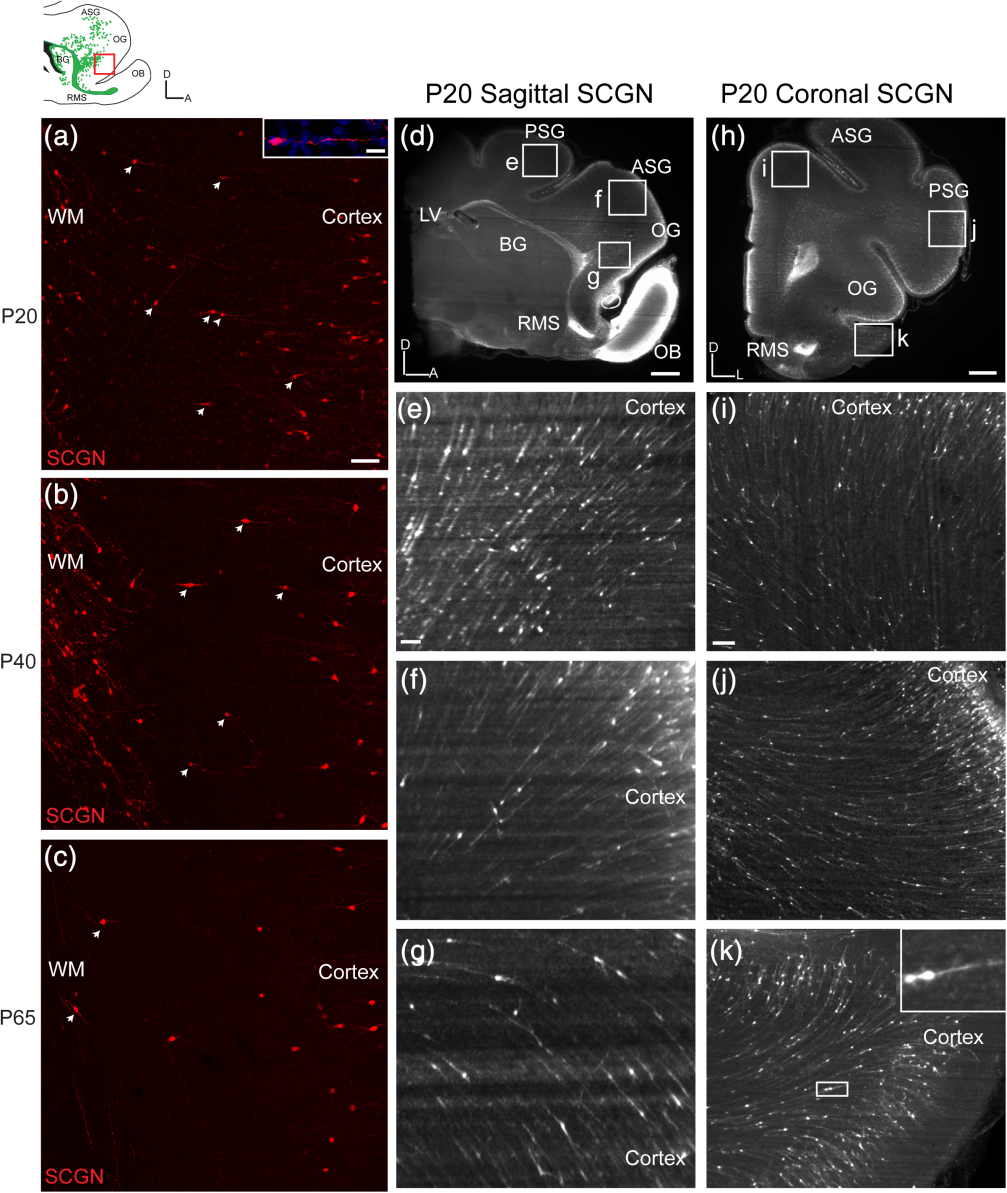
With iDISCO, SCGN+ cells appear to transition between white matter streams and cortex. (a) P20 ferret MMS SCGN+ cells are observed outside the MMS and are oriented toward the prefrontal cortex. (b) At P40, fewer SCGN+ cells are observed outside the MMS oriented toward the prefrontal cortex. (c) At P65, few SCGN+ cells are observed outside the MMS. (d,h) P20 iDISCO cleared sagittal and coronal sections stained with SCGN. (e–g,i–k) Higher resolution images focused on multitude of SCGN+ cells appearing to transition between the white matter streams and cortex. Scale bars a–c = 100 μm. Scale bar inset a = 10 μm. Scale bars d,h = 1 mm. Scale bars e–g,i–k = 100 μm [Color figure can be viewed at http://wileyonlinelibrary.com]

The finding of SCGN+ interneurons oriented toward the prefrontal cortex in ferret provided the unique opportunity to investigate possible contributions to developing cortical circuits. We first stained sagittal sections from P20, P40, P65 and adult ferret with SCGN and looked specifically at the MMS, the stream with the highest degree of co‐expression between DCX and SCGN (Figure 7a). In P20 sections, we were able to observe individual cells with migratory morphology appearing to transition from the white matter stream into the cortex. Cells continued to be observed in this state in P40 and P65 (Figure 7b,c), although the number decreased as predicted by both the DCX maps and quantification. However, considering the density of cells in the MMS, too few cells were observed outside the stream using single section immunohistochemistry to consider any kind of robust cortical contribution. Therefore, we next attempted to clear large tissue sections in order to more accurately visualize how many cells might be transitioning into the cortex.

Using the iDISCO protocol, we cleared 2 mm sections of P20 ferret brain tissue, stained them with SCGN, and imaged them using a light sheet microscope (Figure 7d–k). The number of SCGN+ cells in the white matter made apparent by using iDISCO was significantly greater than that seen using traditional immunohistochemistry. Furthermore, we were able to better visualize the number of SCGN+ cells in the cortex itself. Examining P20 sagittal sections (Figure 7d–g and Movie S4), we were able to observe a seamless transition of SCGN+ cells from the MMS into the prefrontal cortex (Figure 7f,g), as well as a possible transition from the DMS into the posterior sigmoid gyrus (Figure 7e). Examining P20 coronal sections (Figure 7h–k and Movies S1–S3), allowed even better visualization of SCGN+ cells appearing to transition from white matter into the cortex. We next attempted to better characterize SCGN+ cells in the prefrontal cortex.

### 3.7 Cortical SCGN‐expressing interneurons survive into adulthood and express NeuN

**Figure 8:**
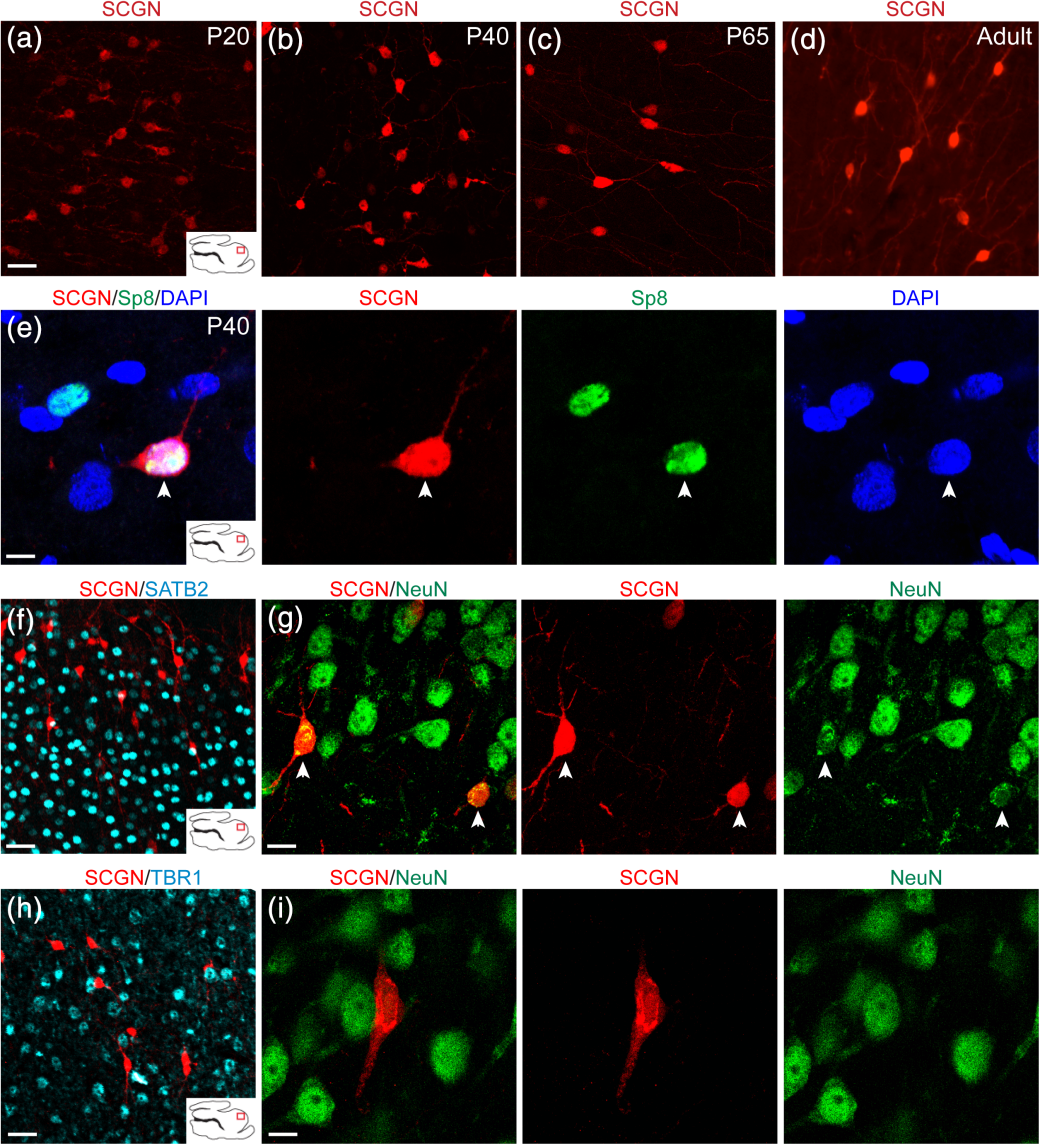
Cortical SCGN+ interneurons survive into adulthood and express NeuN. (a–d) SCGN+ cells in the ASG from P20 to adult. (e) P40 cortical SCGN+ cells continue to express the interneuron marker Sp8. (f,g) P40 SCGN+ interneurons express NeuN in upper cortical layers and have a more uniform arrangement. (h,i) P40 SCGN+ interneurons do not express NeuN in lower cortical layers and are unevenly distributed. Scale bars (a–d) = 10 μm. Scale bar (e) = 7.5 μm. Scale bars f,h = 40 μm. Scale bars g,i = 10 μm [Color figure can be viewed at http://wileyonlinelibrary.com]

In order to better characterize the fate of P20 SCGN+ cortical cells, we stained sagittal sections with SCGN at P20, P40, P65, and adult (Figure 8a–d). Focusing on the ASG, that is, the possible destination of the MMS in the prefrontal cortex, we observed that SCGN+ cells in the cortex were more numerous, and possessed smaller soma, at P20 and P40 (Figure 8a,b). By P65, SCGN+ cells were less numerous and had larger soma, and were observed with multiple neurites (Figure 8c). SCGN+ cortical cells continued to be observed into adulthood and were also observed with longer and more branched neurites (Figure 8d). Cortical SCGN+ cells expressed Sp8 both in P20 (data not shown) and P40 (Figure 8e), suggesting an interneuron identity like their MMS counterparts. We next looked at distribution of SCGN+ cells throughout the developing cortex. Using SATB2 to mark upper cortical layers, we observed a more laminar distribution in the upper layers (Figure 8f). SCGN+ cells in the upper cortical layers co‐expressed NeuN (Figure 8g). Using TBR1 to mark lower cortical layers, we observed a more unorganized distribution (Figure 8h). These cells did not co‐localize with NeuN (Figure 8i). This could imply either that SCGN+ cells in lower cortical layers are migrating to upper layers before maturing, or if they are arriving later, they have yet to express NeuN.

### 3.8 SCGN+ cells exist in the white matter into adulthood

**Figure 9:**
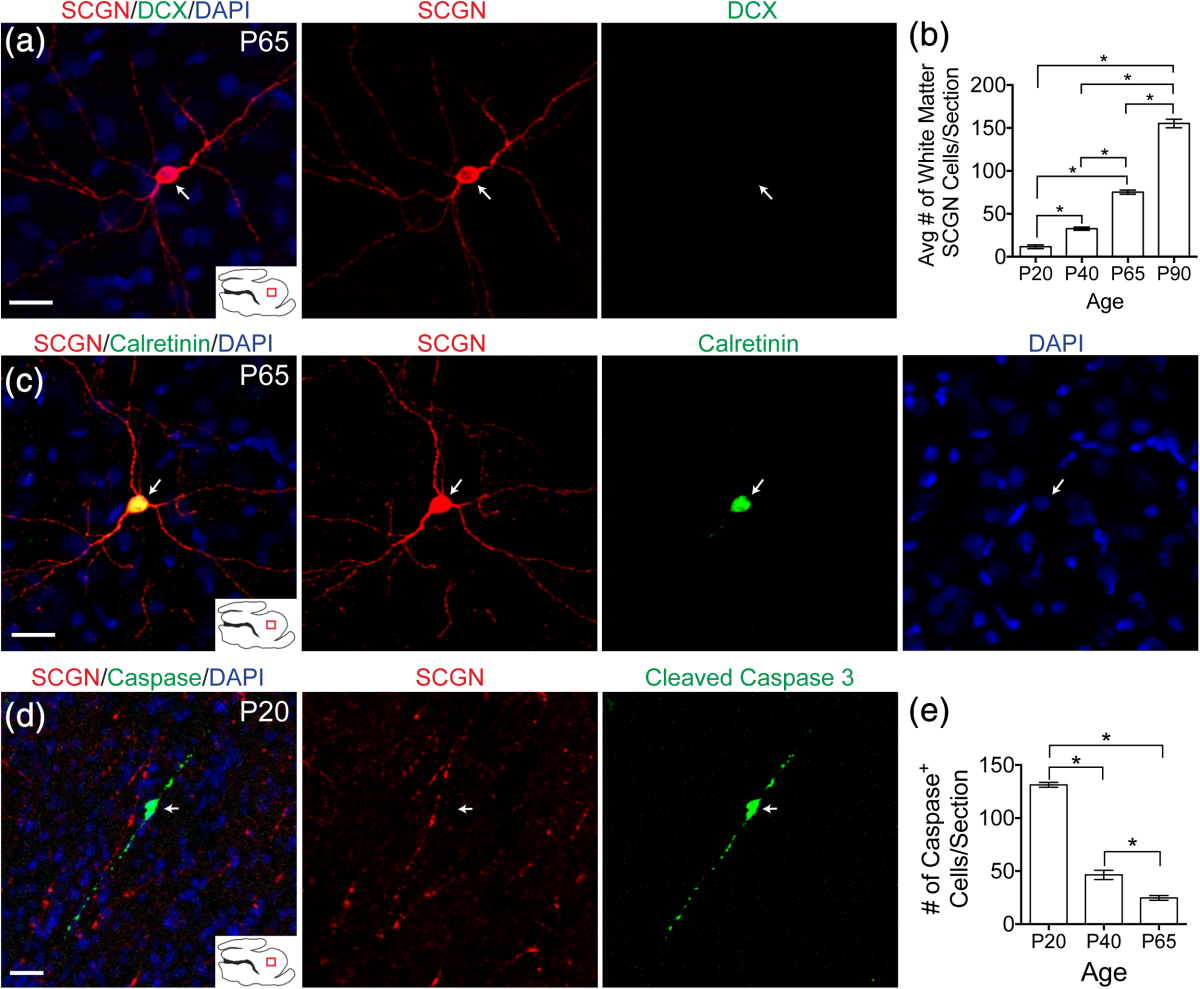
SCGN+ cells exist in the white matter into adulthood. (a) P65 white matter SCGN+ cells with mature morphology do not express DCX. (b) Quantification of number of SCGN+ cells with mature morphology from P20 to P90. (c) White matter SCGN+ cells with mature morphology co‐localize with calretinin. (d) Cleaved caspase 3 signal in the white matter does not co‐localize with SCGN. (e) Quantification of the number of caspase+ cells in the white matter from P20 to P65. Scale bars a,c,d = 20 μm [Color figure can be viewed at http://wileyonlinelibrary.com]

SCGN+ cells were observed in the white matter at all ages studied. Starting at P20, but most apparent at P65, cells could be observed with a mature, nonmigratory, morphology, that is, a large soma and multiple neurites (Figure 9a). These cells also did not co‐express DCX. The number of these mature looking cells continued to increase in the white matter between P20 and P90 (Figure 9b). Many of them expressed the marker calretinin (Figure 9c). To see if these cells underwent apoptosis as part of their fate, cleaved caspase 3 staining was performed. Cleaved caspase 3 signal did not co‐localize with mature white matter SCGN+ cells (Figure 9d). Furthermore, as the number of white matter SCGN+ cells increased significantly from P20 to P90, the number of observed Caspase+ cells decreased significantly (Figure 9e), suggesting that the majority of these cells will survive in the white matter.

## 4 DISCUSSION

The ferret has been used as a model for studying CNS development for many years due to its gyrencephalic cortex and extended postnatal brain development (Chapman, Zahs, & Stryker, 1991); (Finney, Stone, & Shatz, 1998); (Penn, Riquelme, Feller, & Shatz, 1998). Here we show that, similar to human (Sanai et al., 2011); (Paredes et al., 2016), the postnatal ferret brain contains robust streams of cortically oriented young interneurons. We also find evidence for a novel stream oriented toward the posterior cortex that has not been described in human. Finally, we find persistent expression of SCGN, both in interneurons oriented toward, as well as in, the cortex, a finding which has been observed in human, but not rodents. Persistent SCGN expression supports the view that such cells permanently invest the cortex and potentially make functional contributions.

### 4.1 Ferrets possess multiple postnatal collections of young interneurons

Ferrets contain a rostromedial collection of young interneurons that branches off the RMS and is oriented toward the anterior sigmoid gyrus and the orbital gyrus, that is, the ferret prefrontal cortex. Between P20 and P40, the ferret RMS‐like structure condenses, and cells cluster together. This stream then continues past P65, until disappearing by P90. All of these characteristics are similar to the reported MMS in human (Sanai et al., 2011). In human between term and 12 months of age, the equivalency of P90 ferret, a MMS of neurons branches off the RMS and supplies calretinin+ interneurons to the ventromedial prefrontal cortex. The stream also transitions from being widely dispersed at earlier ages to being tightly condensed clusters at later stages.

Ferrets also contain a dorsal collection of young interneurons that potentially originate at a cluster of Dcx+ cells surrounding the midpoint of the lateral ventricles. Their origin is inferred based on their proximity to these clusters, as well as similar timing in the decrease in number of cells in both areas. The stream is contained within the white matter and is oriented radially toward the posterior sigmoid gyrus. The posterior sigmoid gyrus is thought to be equivalent to human anterior cingulate gyrus, a midline region that is a part of the default mode network and is correlated with an individual's ability to mentalize (Apps & Ramnani, 2014). It was recently shown that the human ACC continued to receive migrating interneurons until the first 6 months of postnatal life (Paredes et al., 2016). These interneurons differentiated into a variety of subtypes with the majority of them expressing neuropeptide Y and calretinin. In the ferret brain, we did not observe the expression of neuropeptide Y, calretinin, PV, or SST in DCX+ cells in this stream (data not shown). We did observe Sp8 and SCGN expression, which may provide clues as to the subtypes these cells may ultimately become.

Finally, we found that ferrets contain a posterior collection of young interneurons that appears to originate at a cluster of doublecortin positive cells at the most caudal tip of the lateral ventricles. The stream is contained within the white matter and is oriented toward the occipital cortex. It is observed until P40 and has disappeared by P65. This implies that young interneurons may continue to be added to occipital circuits until P65, and may contribute to the opening of a critical period for vision. It also implies that humans may have a similar, and as yet undiscovered, stream as well.

### 4.2 Characteristics of DCX+ cells in putative migratory streams

Young interneurons in the putative ferret postnatal streams express several markers associated with an origin in the ventral pallium (Ma et al., 2012; Raju et al., 2018). We found they express Sp8 and SCGN to varying degrees, two markers of interneurons shown to be expressed in cells originating in the caudal ganglionic eminence (CGE) and lateral ganglionic eminence (LGE). The DCX+/SCGN+ cells do not express markers or transcription factors associated with the medial ganglionic eminence, such as PV, SST, or Nkx2.1 (data not shown). Interestingly, we found that nearly all of the late migrating DCX+ cells expressed SCGN, a calcium binding protein (Mulder et al., 2009; Rogstam et al., 2007). Previously, secretagogin had been observed in neurons in multiple structures in the rodent brain, such as the RMS, olfactory bulb, hypothalamus, dentate gyrus and amygdala, but not cortex (Kosaka & Kosaka, 2013; Mulder et al., 2009, 2010; Romanov et al., 2015). Recently, it was shown that human cortex contains SCGN+ interneurons, and forced expression in mice increases neurite outgrowth (Raju et al., 2018). The discovery that the ferret also contains SCGN+ interneurons, both in the white matter and cortex, that persist into adulthood, implies that SCGN+ interneurons may represent a change in cortical development found only in gyrencephalic brains whose function and circuit contributions can be further studied using ferret as a model.

Future work is needed to determine the fate of postnatal interneurons in ferret. In the human, the DMS (ARC) and MMS appear to contribute neuropeptide Y and calretinin+ interneurons to developing cortical circuits in the ACC and VMPFC, respectively. In the ferret, interneurons in postnatal streams are cortically oriented. We found that they persist until various time points depending on region and appear to be transitioning from white matter to cortex. Furthermore, persistent cortical SCGN expression, observed into adulthood, suggests that SCGN+ cells from the streams that appear to be exiting the white matter could potentially invest the cortex. In human, SCGN+ cortical interneurons are also observed in the upper layers. We observed SCGN+ cells in both upper and lower cortical layers. However, the cells in the upper layers were more organized into a laminar distribution and co‐localized with NeuN. In contrast, SCGN+ cells in the lower layers were more randomly distributed and did not co‐localize with NeuN. This could imply that cells in the lower layers are migrating into the upper layers. It could also imply that these cells are late‐arriving and have yet to mature and express NeuN.

SCGN+cells were also observed throughout development in the white matter, where they develop extensive neurite branching and express calretinin. Persistent, functional, white matter interneurons have been reported in rodents (von Engelhardt, Khrulev, Eliava, Wahlster, & Monyer, 2011) and both young and older rhesus monkeys (Mortazavi, Wang, Rosene, & Rockland, 2016). Some of the interneurons in the white matter may undergo apoptosis. It has previously been reported that up to 30% of interneurons undergo apoptosis (Southwell et al., 2012). However, caspase staining did not correlate with SCGN as the number of mature SCGN+ white matter interneurons increased while the number of caspase+ white matter cells decreased. If the majority of SCGN+ cells were undergoing apoptosis, it would be expected that the number would better correlated with the pattern of caspase+ cells.

The finding of putative streams of DCX+ interneurons throughout the developing ferret brain suggests that this may be a common developmental phenomenon in some species, possibly related to brain size or gyrification. Perinatal injuries might therefore disproportionately affect the circuits that may be supplied by these cells. A continued addition of late postnatal interneurons would undoubtedly affect the excitatory‐inhibitory balance of those circuits. We conclude that the extended postnatal development of the ferret CNS offers a suitable model to study postnatal interneurons for possible migration and integration into existing circuits. Further studies are needed to test roles for late postnatal interneurons and how their migration and or circuit integration could be impacted by brain injury.

## CONFLICT OF INTEREST

The authors state no conflict of interest.

## AUTHOR CONTRIBUTIONS

All authors had full access to all of the data in the study. Study concept and design: J.E., S.F.S., D.R.; Acquisition of data: J.E., S.M.; Analysis and interpretation of data: J.E., S.F.S., D.R.; Writing of the manuscript: J.E., S.F.S., D.R.; Critical revision of the manuscript: J.E., S.F.S., P.M., D.R.

## Supporting information


**Supplemental Movies 1,2,3. SCGN+ cells appear to transition smoothly from white matter collections into cortex.** Ferret P20 brain was cut into 2 mm thick coronal sections, cleared using iDISCO protocol, stained with SCGN, and imaged on light sheet microscope. Region in Movie 1 corresponds to Figure 7i. Region in Movie 2 corresponds to Figure 7j. Region in Movie 3 corresponds to Figure 7k.

**Supplemental Movie 4 SCGN+ cells appear to transition smoothly from white matter collections into cortex.** Ferret P20 brain was cut into 2 mm thick sagittal section, cleared using iDISCO protocol, stained with SCGN, and imaged on light sheet microscope. Region corresponds to anterior sigmoid gyrus, seen in Figure 7f.
